# A *TNF* Variant that Associates with Susceptibility to Musculoskeletal Disease Modulates Thyroid Hormone Receptor Binding to Control Promoter Activation

**DOI:** 10.1371/journal.pone.0076034

**Published:** 2013-09-19

**Authors:** Endre Kiss-Toth, Edward Harlock, Darren Lath, Thomas Quertermous, J. Mark Wilkinson

**Affiliations:** 1 Department of Cardiovascular Science, University of Sheffield, Sheffield, United Kingdom; 2 Department of Human Metabolism, University of Sheffield, Sheffield, United Kingdom; 3 Division of Cardiovascular Medicine and Cardiovascular Research Institute, Stanford University, Stanford, California, United States of America; Harvard Medical School, United States of America

## Abstract

Tumor necrosis factor (TNF) is a powerful pro-inflammatory cytokine and immuno-regulatory molecule, and modulates susceptibility to musculoskeletal diseases. Several meta-analyses and replicated association studies have implicated the minor ‘A’ variant within the TNF promoter single nucleotide polymorphism (SNP) rs361525 (-238A/G) as a risk allele in joint related disorders, including psoriatic and juvenile idiopathic arthritis, and osteolysis after joint arthroplasty. Here we characterized the effect of this variant on TNF promoter function. A transcriptional reporter, encoding the -238A variant of the TNF promoter, resulted in 2.2 to 2.8 times greater transcriptional activation versus the ‘G’ variant in murine macrophages when stimulated with pro-inflammatory stimuli. Bioinformatic analysis predicted a putative binding site for thyroid hormone receptor (TR) for the -238A but not the -238G allele. Overexpression of TR-α induced promoter expression 1.8-fold in the presence of the ‘A’ allele only. TR-α expression both potentiated and sensitized the -238A response to LPS or a titanium particulate stimulus, whilst siRNA knockdown of either *THRA* or *THRB* impaired transcriptional activation for the -238A variant only. This effect was independent of receptor-ligand binding of triiodothyronine. Immunohistochemical analysis of osteolysis interface membranes from patients undergoing revision surgery confirmed expression of TR-α within osteoclast nuclei at the resorption surface. The ‘A’ allele at rs361525 confers increased transcriptional activation of the TNF promoter and influences susceptibility to several arthritic conditions. This effect is modulated, at least in part, by binding of TR, which both sensitizes and potentiates transcriptional activation of the ‘A’ variant independent of its endogenous ligand.

## Introduction

Tumor necrosis factor (TNF) is a pleiotropic cytokine that has powerful pro-inflammatory and immuno-regulatory functions. TNF plays an important role in the pathogenesis of musculoskeletal disease, and its blockade has become a major therapeutic tool in their treatment [1]. Aseptic loosening due to wear particle-induced osteolysis is the most common cause of joint prosthesis failure requiring revision surgery [2]. TNF plays a pivotal role in osteolysis, inducing osteoclastogenesis by both RANK/RANKL dependent and independent pathways [3-5]. TNF p55 receptor null transgenic mice show resistance to wear particle-induced bone resorption [6], and anti-TNF therapy using Etanercept reduces titanium particle-induced osteoclastic bone resorption both *in-vitro* and *in-vivo* [7,8].

The gene encoding TNF is located within the highly polymorphic major histocompatibility complex region on chromosome 6p21, 3 [9]. Several meta-analyses have linked carriage of the minor ‘A’ allele of the G/A single nucleotide polymorphism (SNP) at the -238 position (rs361525, global minor allele frequency 5% in 1000 Genomes dataset) of the TNF promoter with increased susceptibility to musculoskeletal diseases, including Behçets disease [10], systemic lupus erythematosus [11], juvenile idiopathic arthritis [12], and psoriatic arthritis [13]. We have previously shown that rs361525 is also a susceptibility locus for osteolysis in patients after total hip arthroplasty [14]. This association has been replicated in an independent population by Gallo et al [15]. In both studies carriage of the ‘A’ allele associated with both increased susceptibility to, and severity of osteolysis. In support of a functional role in-vivo for the ‘A’ variant at this site Sapey et al. [16] found in a longitudinal study of bronchitis patients that those carrying rs361525 had more chronic bronchitis and a greater annual decline in lung function. Bioactive TNF protein levels in their airway secretions were 100-fold higher than non-carriers, and their lung secretions contained more IL-8 and myeloperoxidase, consistent with greater downstream inflammation.

Genetic variation within a gene promoter can influence gene activity, SNPs in the transcribed region can impact on pre-mRNA stability and can also lead to mutations which alter the ability of protein to bind to its substrates or inhibitors as well as changing sub-cellular localization of proteins, and thereby modulate disease susceptibility [17]. Several transcription factor binding sites exist within the TNF promoter, including NFκB, Ets, NF-AT, AP-1, STAT1, and LITAF. Despite the considerable published literature, the contribution of these regulatory elements to TNF activation and expression is incompletely understood [18], and the functional relevance of rs361525 on TNF promoter activity in the setting of musculoskeletal disease remains unclear.

The aim of this study was to determine the functional effect of the rs361525 variant on TNF promoter transcriptional activation using an *in-vitro* macrophage model, in response to a variety of clinically-relevant stimuli. Clearer understanding of the molecular mechanisms by which this variant confers susceptibility to diseases linked to this locus, including osteolysis, Behçets disease, systemic lupus erythematosus, juvenile idiopathic arthritis, and psoriatic arthritis, may lead to novel therapeutic strategies in their treatment.

## Results

### The ‘A’ variant of rs361525 enhances the responsiveness of TNF promoter to TLR-mediated activation

**Figure 1:**
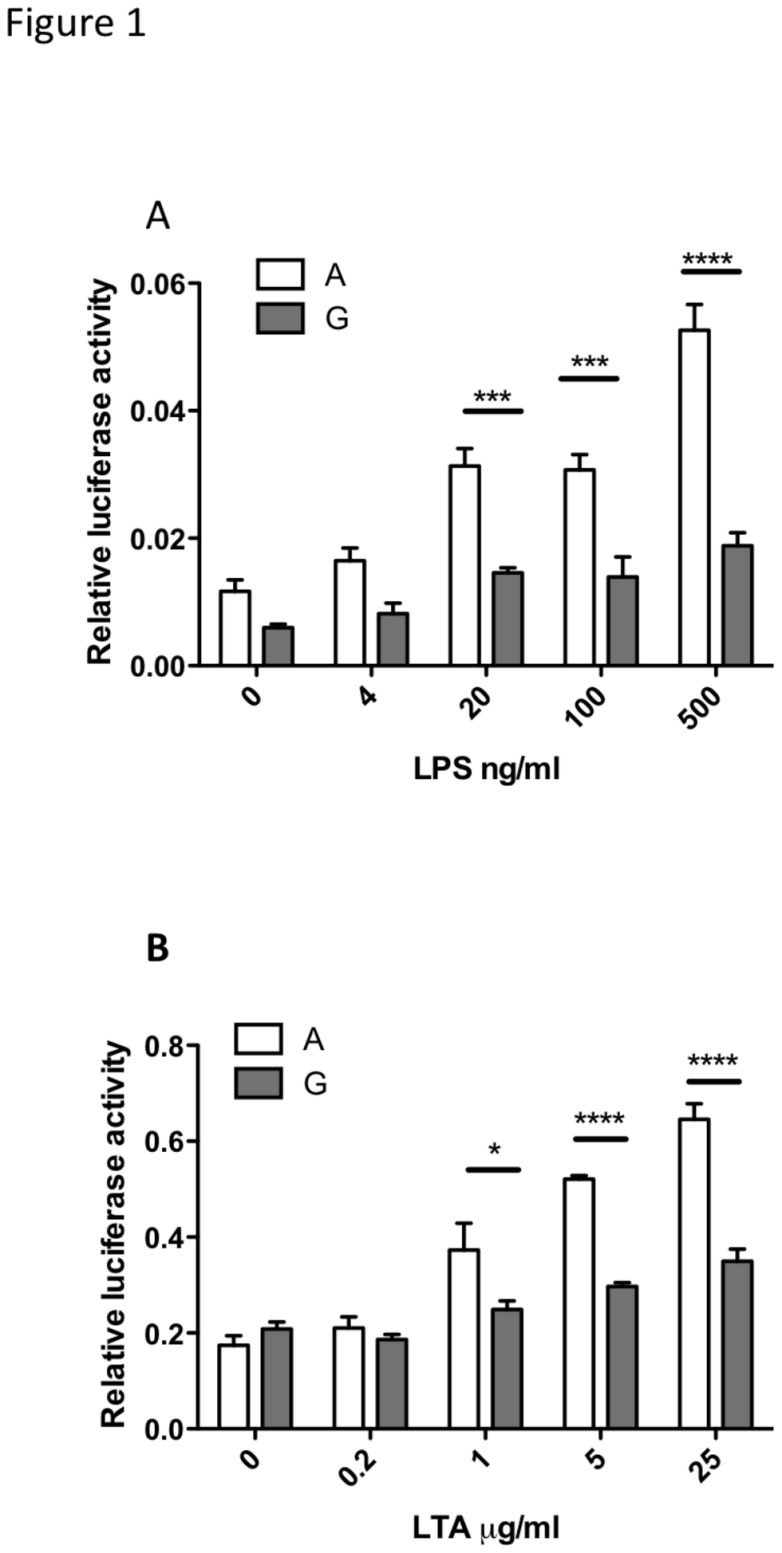
TNF promoter containing the ‘A’ variant of rs361525 has an augmented response to TLR4 and TLR2 ligands, compared to the -238G variant. Raw 264.7 cells were transiently transfected with firefly-luciferase reporters under the control of the ‘A’ or the ‘G’ variants of the TNF promoter, and co-transfected with a constitutive Renilla-luciferase reporter. Transfected cells were stimulated with the stated concentration of lipopolysaccharide (LPS, TLR-4 ligand; panel **A**) or Lipoteichoic acid (LTA, TLR-2 ligand; panel B) for 6 hours. A representative of four experiments is shown. Results were analyzed by two-way ANOVA with a Bonferroni post-test. * p<0.05, *** p<0.001, **** p<0.0001.

In order to test the functional importance of the ‘A’ versus ‘G’ variation in rs361525, luciferase reporters were developed, under the control of the TNF promoter, encoding for each of these variants. In order to rule out the influence of other SNPs, the promoter allele, encoding for the ‘G’ variant at -238 was subjected to sited directed mutagenesis, generating the ‘A’ allele (for the full promoter sequence, see Figure 1). Responses against a range of concentrations for Gram negative (LPS) as well as Gram positive (LTA) bacterial cell wall components were evaluated, using Raw 264.7 cells that were transiently transfected by these reporters (Figure 1). Whilst both TNF promoter variants were induced by these stimuli, the construct containing the ‘A’ variant showed consistently higher response to both LPS and LTA (Figure 1).

### The ‘A’ variant of rs361525 encodes for a putative thyroid hormone receptor (TR) binding site

**Figure 2:**
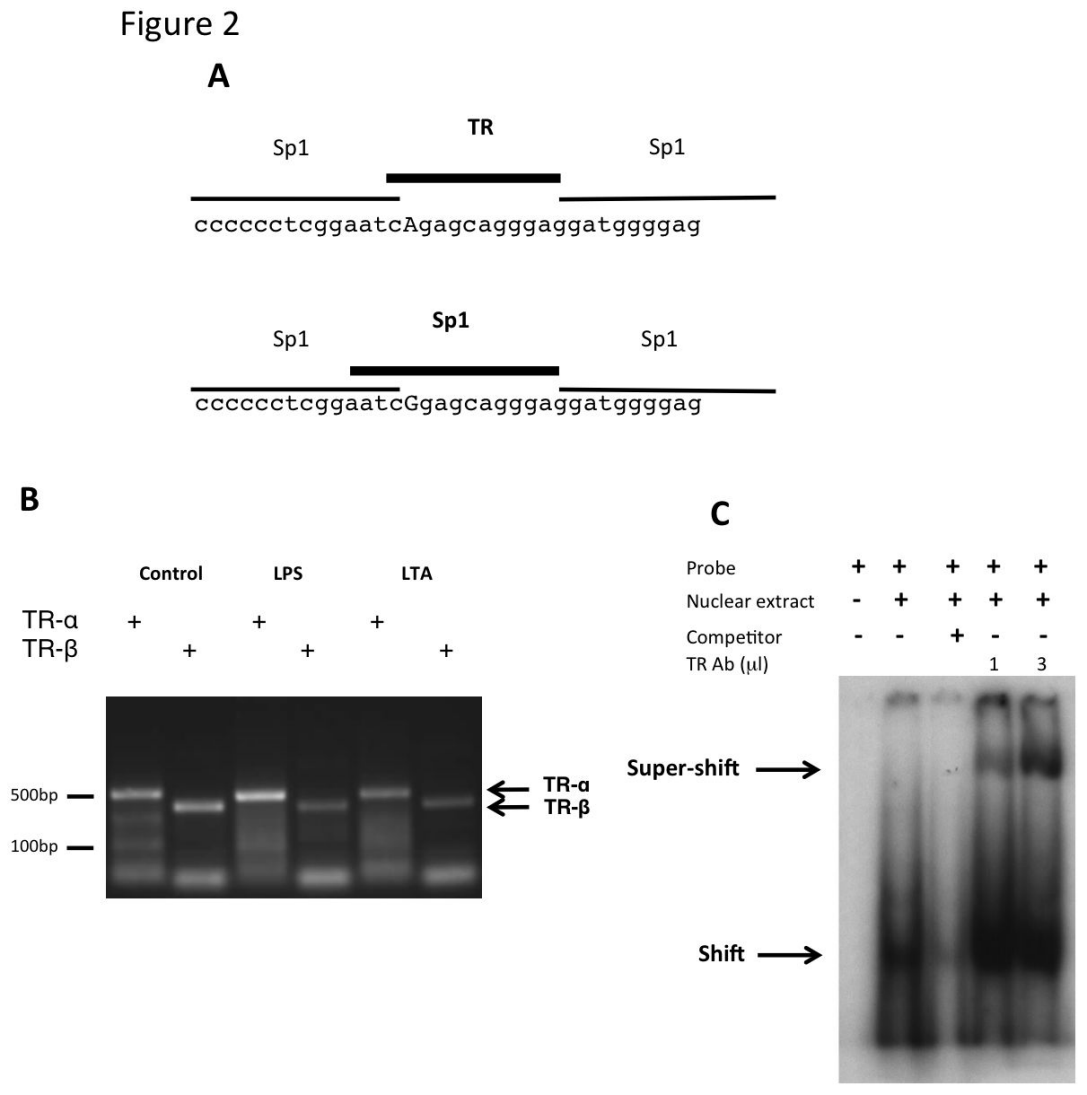
Bioinformatic analysis predicts a putative binding site for thyroid hormone receptors for the ‘A’ variant at rs361525. Sequences surrounding the -238 A/G polymorphism were analysed by AliBaba 2.1 (http://www.gene-regulation.com/pub/programs/alibaba2/index.html) to predict putative allele-specific transcription factor binding sites (Panel A). The polymorphic nucleotides are capitalised and the binding sites spanning this position are highlighted in bold. The expression of *THRA* and *THRB* in Raw264.7 cells was confirmed by RT-PCR (Panel B). Total RNA was purified from control, LPS stimulated (40 ng/ml, 6 hrs) or LTA stimulated (5 µg/ml, 6 hrs) cells, reverse transcribed and expression was detected by gene specific PCR primers (*THRA*: 480 bp product, *THRB*: 430 bp product). EMSA was performed to demonstrate binding of TR to the “A” variant of rs361525 (Panel C). Specificity of binding was ascertained by competition with a 100-fold molar excess of cold DNA fragments added to the reaction mixture (lane 3). For super-shift assay, the nuclear extract were incubated with an anti-TRβ polyclonal antibody.

*In-silico* analysis of the sequence surrounding rs361525 predicted a binding site for thyroid hormone receptor (TR) for the ‘A’ allele and a binding site for Sp1 for the ‘G’ allele (Figure 2A). Next, we characterized the potential involvement of TR in the control of TNF expression in Raw 264.7 macrophage like cells. TR proteins are encoded by two genes, *THRA* and *THRB*, which are expressed in primary macrophages. RT-PCR analysis revealed that both of these genes are expressed in this cell line (Figure 2B), making it a suitable *in vitro* system to investigate the impact of the receptor in TNF expression. Next, we demonstrated that the “A” allele interacts with TR, by analysis with an electrophoretic mobility shift assay (GMSA) (Figure 2C). A radiolabeled DNA probe encoding the "A" allele showed evidence of protein binding when incubated with nuclear extracts, and the specificity of the observed shifted complex was confirmed by competition with excess cold probe. Incubating of the binding reaction in the presence of anti-TRβ polyclonal antibody resulted in a super-shifted complex consistent with binding of the antibody to a TRβ protein-DNA complex.

### Overexpression of TR-α selectively induces the TNF promoter containing the ‘A’ variant of rs361525 and is independent of thyroid hormone binding

**Figure 3:**
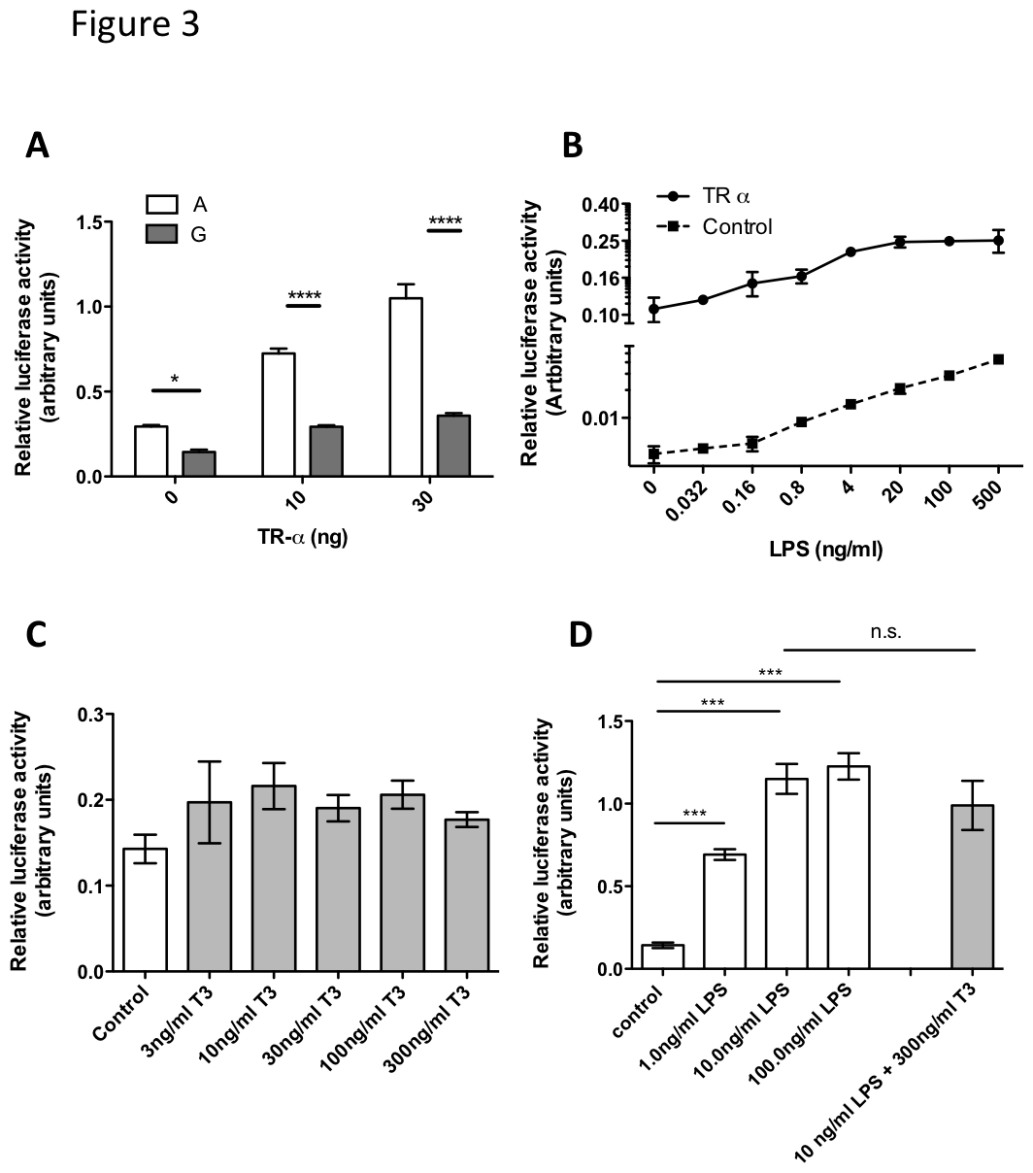
Overexpression of TR-α induces the activation of the TNF promoter containing the ‘A’ variant of rs361525 in synergy with TLR-4 mediated promoter induction, and is independent of thyroid hormone binding. Raw 264.7 cells were transiently transfected with firefly-luciferase reporters under the control of the ‘A’ or the ‘G’ variants of the TNF promoter, at position -238 and co-transfected with the stated amount of TR-α expression plasmid (Panel A). Raw 264.7 cells were transiently transfected with the firefly-luciferase reporter under the control of the ‘A’ variant of the TNF promoter, and co-transfected with 30ng of TR-α expression plasmid (Panel B). Transfected cells were stimulated with the stated concentration of lipopolysaccharide (LPS) for 6 hours. Raw 264.7 cells were transiently transfected with the firefly-luciferase reporter under the control of the ‘A’ variant of the TNF promoter and stimulated with the stated concentration of thyroid hormone (TH) and LPS for 6 hours (panels C and D). Results for panels A and B were analyzed by two-way ANOVA with a Bonferroni post-test, for panels C and D by one-way ANOVA with a Bonferroni post-test. *** p<0.001, **** p<0.0001.

In order to assess the potential interaction between the TR and the TNF promoter in macrophages, Raw 264.7 cells were transiently transfected with the ‘A’ or the ‘G’ variant of the TNF promoter driven luciferase reporter in the presence or absence of the TR-α expression vector. Whilst expression of an increasing dose of TR-α had no effect on the activity of the ‘G’ allele, the reporter driven by the ‘A’ allele was induced in a dose-dependent fashion (Figure 3A). Next, the impact of TR-α expression on the LPS induced activation of the TNF -238A allele was investigated. Induction of the reporter was augmented as well as sensitized in the presence of co-expressed TR-α Figure 3B). Half max. reporter activation was achieved at ~0.5ng LPS in the presence of co-expressed TR-α versus 10ng LPS in controls, suggesting that binding of this protein to the TNF promoter may contribute to the inducibility, as well as to the sensitivity, of TNF activation.

Thyroid hormone receptors are localized in the cell nucleus. However, they are thought to be complexed with co-repressor proteins in the absence of ligand binding, thus unable to activate their targets in most cases. Therefore, the impact of thyroid hormone (as the biologically active hormone triiodothyronine/T3) on the activity of TNF promoter was assessed in Raw 264.7 cells. Cells were transiently transfected with the TR responsive reporter driven by the -238A allele and the effect of increasing dose of T3 on promoter activity (Figure 3C) and the impact on LPS induced reporter activation (Figure 3D) was investigated. Our results showed that T3 does not influence activation of the TNF promoter, suggesting that the action of TR encoded by the -238A variant is independent of its endogenous ligand.

### siRNA knockdown of TR mRNA selectively impairs the activity of the TNF promoter containing the ‘A’ variant of rs361525

**Figure 4:**
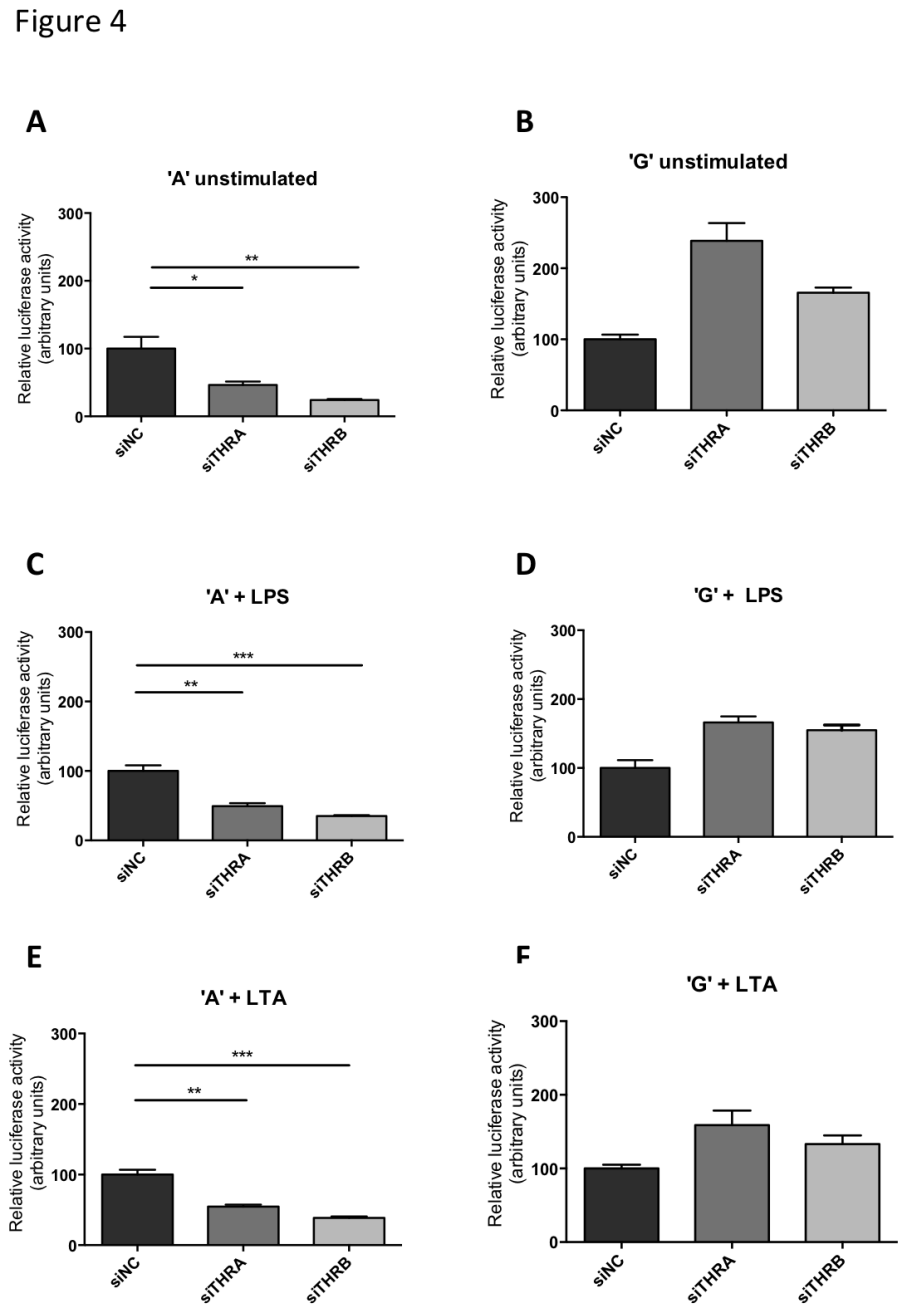
Knockdown of TR-α and TR-β mRNA impairs basal and TLR induced expression of the TNF promoter containing the ‘A’ variant of rs361525. Raw 264.7 cells were transiently transfected with firefly-luciferase reporters under the control of the ‘A’ (panels A-C) or the ‘G’ (panels D-F) variants of the *TNF* promoter and co-transfected with non-targeting (siNC), *THRA* or *THRB* targeting siRNAs, respectively. Transfected cells were stimulated with the stated concentration of lipopolysaccharide (LPS, panels B and E) or Lipoteichoic acid (LTA, panels C and F) for 6 hours. Results were analyzed by one-way ANOVA with a Bonferroni post-test. * p<0.05, ** p<0.01, *** p<0.001.

Next, we wished to clarify whether TR expression is necessary for the full activation of TNF by LPS or LTA. Raw 264.7 cells were transfected with luciferase reporter driven by either the -238A or the -238G variant and induced reporter activity was measured. The impacts of pools of four siRNAs against *THRA* and *THRB* versus non-targeting (siNC) on reporter activity were compared under basal and stimulated conditions (Figure 4). Efficiency of the siRNA knockdown was verified by qRT-PCR (Figure 2). In agreement with the *in-silico* predictions and our TR-α over-expression results, selective inhibition of -238A transcriptional activity was observed in TR knockdown for basal expression, and following LPS or LTA stimulation (Figure 4A, C and E). Such inhibition was not observed for the construct containing the ‘G’ allele (Figures 4B, D and 4F). Indeed, a small increase in the activity of the ‘G’ allele was observed in response to siTR treatment. Whilst the reasons for this are unclear, it is possible in addition to activating TNF expression via the -238 ‘A’ allele, TR proteins may also interact with other factors, that are (directly or indirectly) involved in the control of TNF expression and that the net effect of some of these interactions may be inhibitory.

### Titanium particles induce a TR-dependent activation of the TNF promoter containing the ‘A’ variant of rs361525

**Figure 5:**
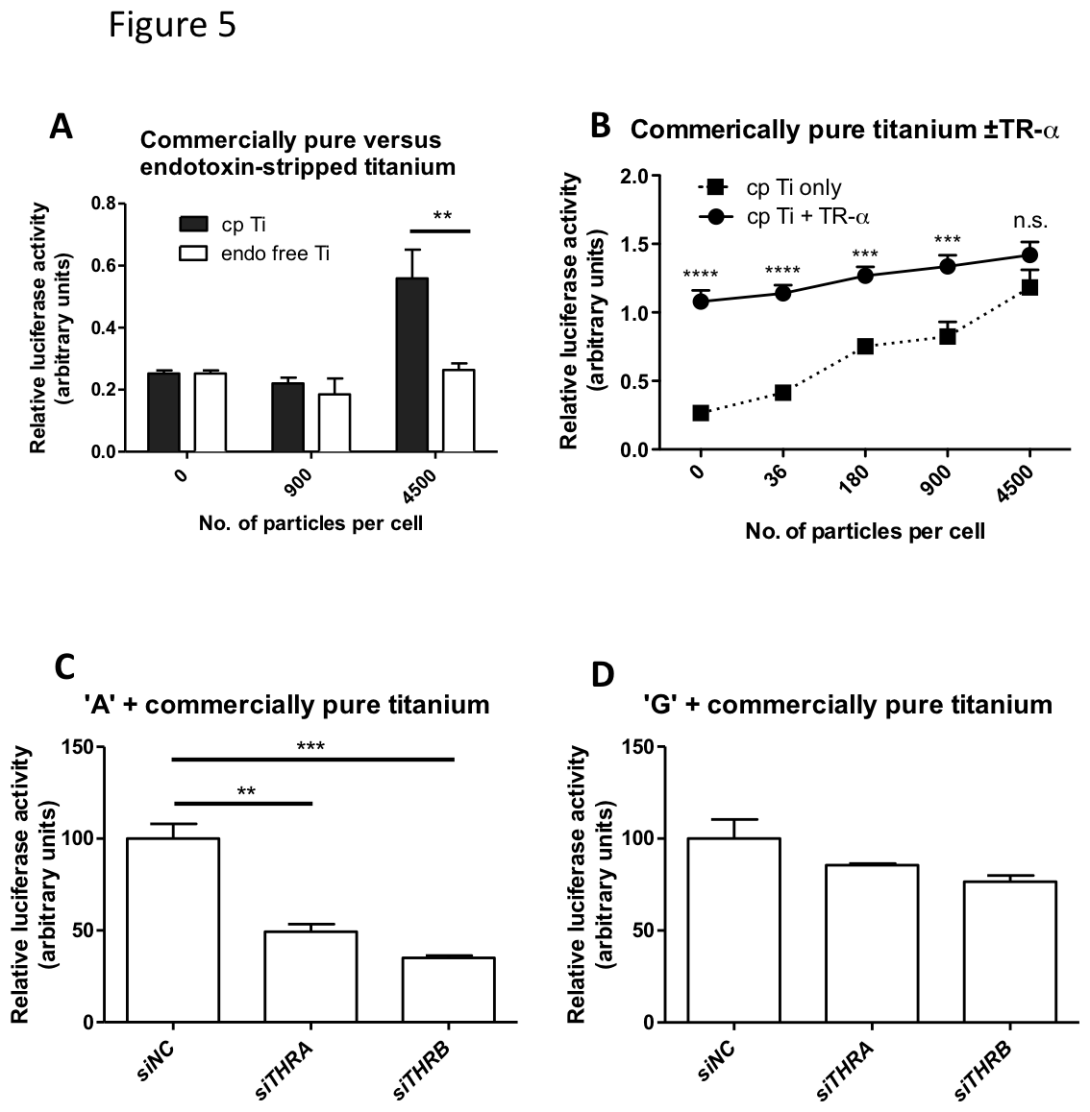
Titanium particles bind LPS and can activate TNF promoter containing the ‘A’, but not the ‘G’ variant of rs361525. Raw 264.7 cells were transiently transfected with the firefly-luciferase reporter under the control of the ‘A’ (panels A-C) or the ‘G’ variant (panel D) of the *TNF* promoter, and co-transfected with 30ng of TR-α expression plasmid (panel B) or with non-targeting (siNC), *THRA* or *THRB* targeting siRNAs, respectively (panels C-D). Transfected cells were stimulated with the stated concentration of Ti particles (panels A-B) or 4500 particles/cell (panels C-D) for 6 hours. Results were analyzed by one-way ANOVA with a Bonferroni post-test. ** p<0.01, *** p<0.001.

In periprosthetic osteolysis macrophage activation results from ingestion of particulate wear debris from the prosthesis surfaces, an effect that is potentiated by absorption of bacterial debris onto the particle surface [19,20]. We investigated the interaction between titanium particles, TNF promoter activation and the TR. In line with previous studies [21], commercially pure titanium particles, but not endotoxin-stripped ones, were able to activate the luciferase reporter driven by the -238A allele of the TNF promoter (Figure 5A). The contribution of the TR to this was demonstrated by a sensitized response in the presence of overexpressed TR (Figure 5B), as well as by an impaired activation of the -238A but the -238G allele in cells where TR-α or β were knocked down using si*THRA* and si*THRB* (Figure 5C vs. D).

### TR-α is expressed in clinical osteolysis membrane-bone interface tissue retrieved from patients

**Figure 6:**
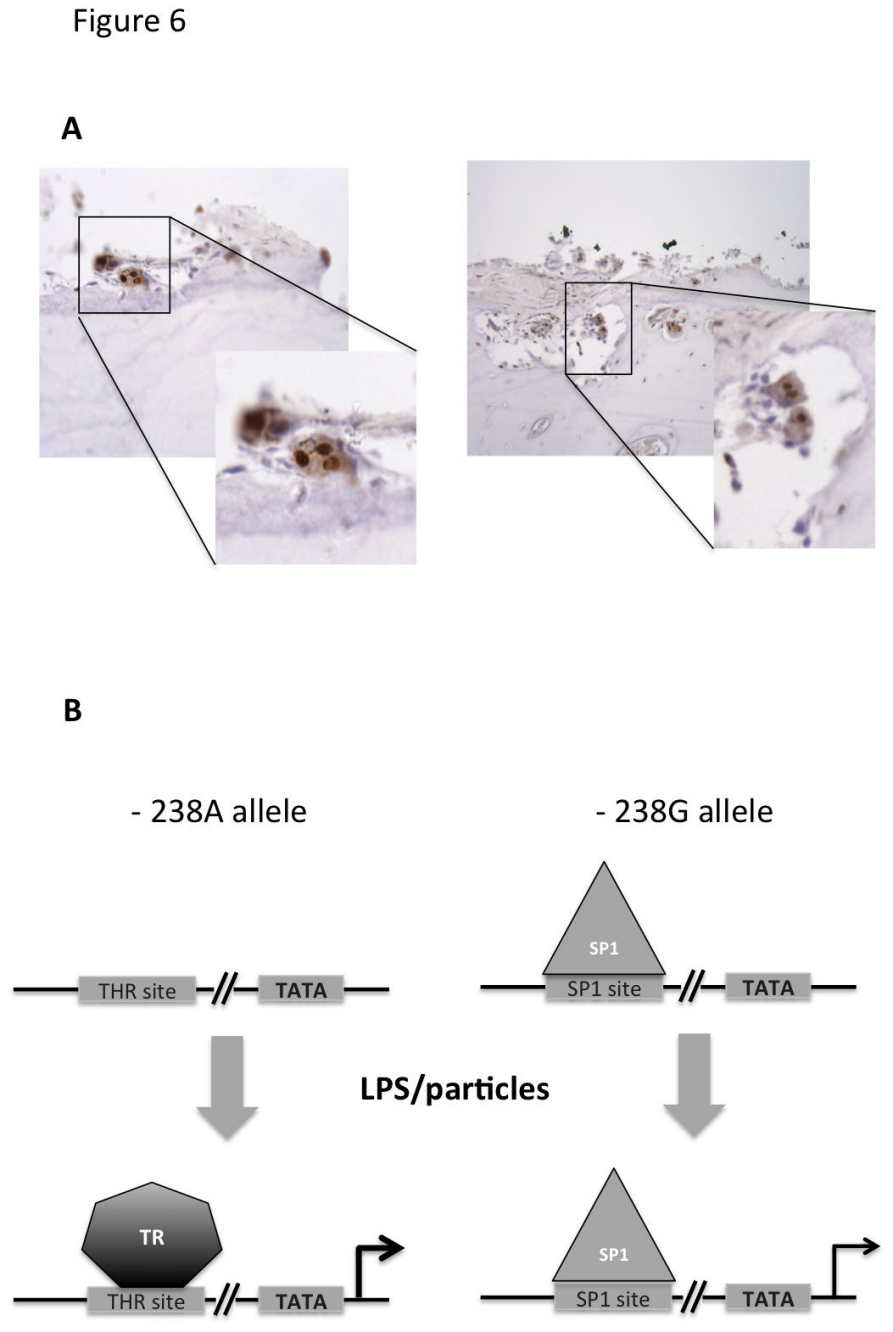
TR-α is expressed in osteoclasts at the osteolysis-membrane interface in patients with failed hip replacement and proposed mechanism of TR action on TNF promoter activation. Panel A: Photomicrographs of osteolysis interface membrane-bone sections from 2 representative patients (total n=6) and stained for anti-thyroid TR-α1 and TR-α2, demonstrating nuclear staining of osteoclasts lying on bone surfaces. Panel B: LPS induced activation of the -238A TNF promoter allele is facilitated by the binding of TR. In contrast, the SP1 site in the -238G allele could be occupied by SP1 even in the absence of TLR stimulus, resulting in a less inducible expression of the TNF promoter.

Macrophage/osteoclasts are key players in the progression of osteolysis, in part due to their ability to express inflammatory cytokines, including TNF. Membrane-bone biopsied previously retrieved from patients undergoing revision surgery for confirmed aseptic loosening were stained for TR-α, to confirm whether the TR is expressed in clinically relevant tissues in a disease that is modulated by allelic variance at rs361525. Immuno-staining staining of the interface tissues confirmed the presence of TR-α protein was expressed within the nuclei resorbing osteoclasts at the bone surface (Figure 6A).

## Discussion

Previous replicated studies have implicated the *TNF* locus rs361525 in susceptibility and severity of many musculoskeletal diseases. Using *in-silico* analysis we identified a TR binding site in the presence of the ‘A’ but not the ‘G’ variant at this locus. Subsequent assays demonstrated that overexpression of the TR-α, which predominates in bone [22], increased transcriptional activation of the ‘A’ variant of the TNF promoter through both the sensitization and potentiation of the signal. This effect was observed with stimulation using both common toll-like receptor agonists and a particulate stimulus relevant to periprosthetic osteolysis, and was independent of the receptor’s binding to its endogenous ligand. Finally, we confirmed that TR-α protein is expressed in the nuclei of osteoclasts at resorption surfaces in biopsies of osteolytic tissue taken from patients undergoing revision arthroplasty surgery.

Studies in primary human monocytes have shown that inflammatory cytokine responses in patients susceptible to osteolysis are greater than those in patients who do not demonstrate this susceptibility [23], and that TNF induction shows marked inter-individual variation [24]. Raabe et al., in a series of experiments using LPS-stimulated RAW 264.7 cells showed that control of TNF production in this system is thought to occur primarily at the transcriptional and not the translational level [25]. Bayley et al. constructed a series of mutants in a large 1176bp fragment of the TNF promoter which replaced the SNPs at -376, -308, -244 and -238 with 10bp link scan sequences to abrogate binding motifs, and found that the -244/-238 region, but not the -308 and -376 regions, modulate transcriptional regulation in some cell lines [26].

Both the α and β isoforms of the TH are expressed in bone, and knockout mice for *THRA* and *THRB* have a bone phenotype. TR-α knockout have delayed bone maturation, but a high bone mass phenotype on aging, whilst TR-β knockout mice have anabolic actions during growth but rapid bone loss in maturity [27]. Thyroid hormone receptors mediate the bone resorptive action of TH in adult mice, and this effect is mediated by the TR-β [28]. Our data indicates that TR-α may modulate a bone loss phenotype through enhanced *TNF* induction independent of TH. In contrast, Sp1 binding sites are occupied by a family of ubiquitously expressed transcription factors, that control of expression for “house-keeping” genes and hormones and is thought to play a role in the regulation of early development and metabolism [29], in-keeping with our observation that the ‘G’ allele at the rs361525 locus is relatively non-inducible. Our findings suggest a functionally relevant novel mechanism for modulating transcriptional activation of the TNF promoter (Figure 6B), and that TR may modulate responses to agonists signaling through TLR-2, TLR-4, and the inflammasome, which are important inducible pathways in inflammatory arthritis and periprosthetic osteolysis [30].

Previous studies describing the function of polymorphisms within the TNF promoter have produced inconsistent results [31,32]. *TNF* lies within a highly polymorphic region within the MHC that contains many inflammatory and immune response related proteins [33]. There is also extensive linkage disequilibrium between SNPs across the MHC. Such allelic confounders and cell-type and stimulus specific effects are likely to contribute to the variable findings. We aimed to overcome such effects by investigating the impact of a single polymorphism in “isolation”, using plasmid based, transcriptional reporters. We employed several, physiologically relevant TLR agonists in combination with overexpression and knockdown of endogenous TR’s to generate a complementary set of experiments with high internal consistency. However, these experiments also have limitations. Firstly, we used an established cell line rather than primary cells. However, whilst osteolysis is principally a macrophage-driven process, thus making the Raw 264.7 macrophage cell line an appropriate model to use, the macrophages within a joint are highly differentiated; there are multinucleate osteoclasts, resident tissue macrophages and monocytes all of which are different from an *in-vitro* murine macrophage. Also, the experiments investigated the impact of human promoter variants in a murine-derived macrophage cell line so the expression activity of the promoter may not be represented as accurately as it would be in a human cell line. Nevertheless, the sequence specificity of transcription factors is unlikely to differ between human and murine systems. Given that the promoter region of the TNF gene is highly variable, it is conceivable that other SNPs, and their impact on the binding of transcription factors may modulate the ability of TR to bind to -238. This could be addressed by an extensive ChIP analysis, which will be the focus of future studies.

In conclusion, we found that the rs361525 locus influences transcriptional activity of *TNF* promoter in response to both TLR agonists and clinical relevant particles. Our data indicate that the ‘A’ allele creates a functionally-active TR binding site. We confirmed that this receptor is expressed within osteoclast cells within osteolytic lesions in arthroplasty patients undergoing revision surgery. These findings may also have functional relevance for other inflammatory joint conditions under TNF control and modulated by rs361525, including ankylosing spondylitis, systemic lupus erythematosus, juvenile idiopathic arthritis, and Behçets disease.

## Materials and Methods

### Ethics

This study was approved by Oxford NRES Research Ethics Committee C, and by Sheffield Musculoskeletal Biobank. The study was conducted according to the principles expressed in the Declaration of Helsinki, and all patients provided written, informed consent prior to participation

#### Cell lines and transient transfections

Raw 264.7 cells were purchased from ATCC (Cat. No: TIB-71, LGC Standards, Teddington, UK) and maintained according to the suppliers’ recommendations. All transfections described in this study were performed in 96 well plates, using three biological replicates. For transient transfection, Dharmafect Duo (Dharmacon, Inc., Chicago, IL) was used, as recommended by the manufacturer (100 ng/well plasmid DNA, including 75 ng reporter DNA (containing a mixture of the TNF promoter-Firefly luciferase and the EF1 promoter-Renilla luciferase reporters) and 25 ng cDNA expression plasmid (or empty vector)/well and 10 pmol siRNA). After transfection, cells were incubated in 100 µl of complete medium (DMEM+10% heat inactivated fetal bovine serum). 16 hours after transfection, agonists were added in 5 µl of PBS to the medium. Cells were lysed 6 hours after stimulation and luciferase assays performed with Dual Luciferase Assay (Promega Corp., Madison, WI). Each experiment was performed at least three times, with similar results.

### Plasmids

Two firefly luciferase reporters (containing either the A or the G allele of rs361525) were used in this study, based on the pGL3.basic vector (Promega Corp.) containing a 691 bp fragment (-585 to +106) of the gene *TNF*. The *TNF* inserts were fully sequenced to confirm that aside from the rs361525 A/G polymorphism, the sequences were identical. An EF1-driven Renilla luciferase reporter was used, as described previously. For overexpression of mouse TR-α, a cDNA clone was isolated and sequenced from a pSPORT6 based custom cDNA expression library. Pools of four siRNAs against mouse thyroid hormone receptor genes *THRA* and *THRB* (Smartpool Target Plus) were purchased from Dharmacon, Inc. and used according to the manufacturer’s recommendation.

## PCR

RT-PCR was used in order to detect expression of *THRA* and *THRB* in Raw 264.7 cells, using standard protocols. Primers to amplify the respective thyroid hormone receptor (TR) cDNAs were:

TR-α: sense 5’- ATGCCGGACGGAGACAAGGTAGAC -3’;
TR-α: antisense 5’- TTGGGCCAGAAGTGCGGAATGTT -3’;
TR-β sense: 5’- AAGTCGGGCTGCCTGAGTTCTA -3’;
TR-β antisense: 5’- TCTAATGGGGCTTCTTCCTTCTAT -3’
Agonists

Lipopolysaccharide (LPS), lipoteichoic acid (LTA) and triiodothyronine (T3) were purchased from Sigma Aldrich (Sigma Aldrich, Cambridge, UK). Titanium particles were supplied as a gift from Dr EM Greenfield (Case Western Reserve University, Cleveland, OH). Two types of particles were used; commercially pure (cp) endotoxin free titanium particles (LPS concentration 1.5 EU/ml), and endotoxin-stripped Ti particles (0 EU LPS/ml). The protocols for preparation of the particles are described in detail elsewhere, as are their size characteristics and ability to induce cytokine production in mononuclear cells [34,35].

### Clinical osteolysis membrane-bone interface tissue retrieval and Immunohistological analysis

Membrane-bone biopsies in cases of periprosthetic osteolysis are routinely retrieved at our institution for diagnostic purposes, and subsequently retained in a clinical histological archive. Sections from 6 anonymized patients were retrieved from the diagnostic archive for this analysis following National Research Ethics Committee and local Biobank approval. Four micron sections were deparaffinized in xylene, hydrated through graded alcohols. Sections were treated with an aqueous solution of 3% H_2_O_2_ to block endogenous peroxidase staining. Antigen retrieval was carried out by trypsin dilution. The sections were incubated with blocking serum followed by overnight at 4°C in primary antibody (rabbit polyclonal anti-thyroid hormone receptor alpha 1+2, Abcam, Cambridge, UK). A secondary goat anti rabbit antibody (Vector Labs Ltd, Peterborough, UK) was added to the sections, followed by an Avidin Biotin Complex solution (Vector Labs Ltd). For visualization, a DAB solution (Vector Labs Ltd) and a haematoxylin counterstain were added to the sections.

### EMSA

Electric Mobility Shift Assay was performed according to the method of Lee et al [36]. Complementary oligonucleotides of the following sequence were synthesized and end labelled with [ϒ^32^P] dATP and polynucleotide kinase prior to annealing: TRAF - 5' -cccccctcggaatcAgagcagggaggatgg, TRAR - 5' - ccatcctccctgctcTgattccgagggggg. Protein extract were isolated from human coronary artery smooth muscle cells, as described previously [36] and analysed by 4% native polyacrylamide gel electrophoresis in 0.5x TBE buffer. Specificity of binding was ascertained by competition with a 100-fold molar excess of cold DNA fragments added to the reaction mixture. For super-shift assay, the nuclear extract was incubated with an anti-TRβ polyclonal antibody (PA1-213, clone TRβ-62, Affinity Bioreagents, Golden, CO) at 4 °C for 2 h before addition to the reaction mixture.

## Supporting Information

Figure S1 **The nucleotide sequence of the promoter region of the TNF gene used in this study has been verified by sequencing.** Polymorphic residues that have been previously been studied by Bayley at all as part of the same haplotype are highlighted (bold, italics, underlined). The -238 A/G variant that was the focus of this work is highlighted in red.
(TIFF)

Figure S2 **Reproducible knockdown of Thra and Thrb was achieved (n=3) in Raw 264.7 cells, as verified by gene specific Taqman qRT-PCR (ABI).** Mean expression values (relative to beta-actin levels) were used and expressed as normalized to control treated (siNC) samples. Experimental conditions were identical to those, used in experiments that are presented in Figures 4-5.
(TIFF)
