# Supplementary figures and images for: A TNF Variant that Associates with Susceptibility to Musculoskeletal Disease Modulates Thyroid Hormone Receptor Binding to Control Promoter Activation
Source: PLoS One. 2013 Sep 19;8(9):e76034. doi: 10.1371/journal.pone.0076034 (PMC3777919; doi:10.1371/journal.pone.0076034)

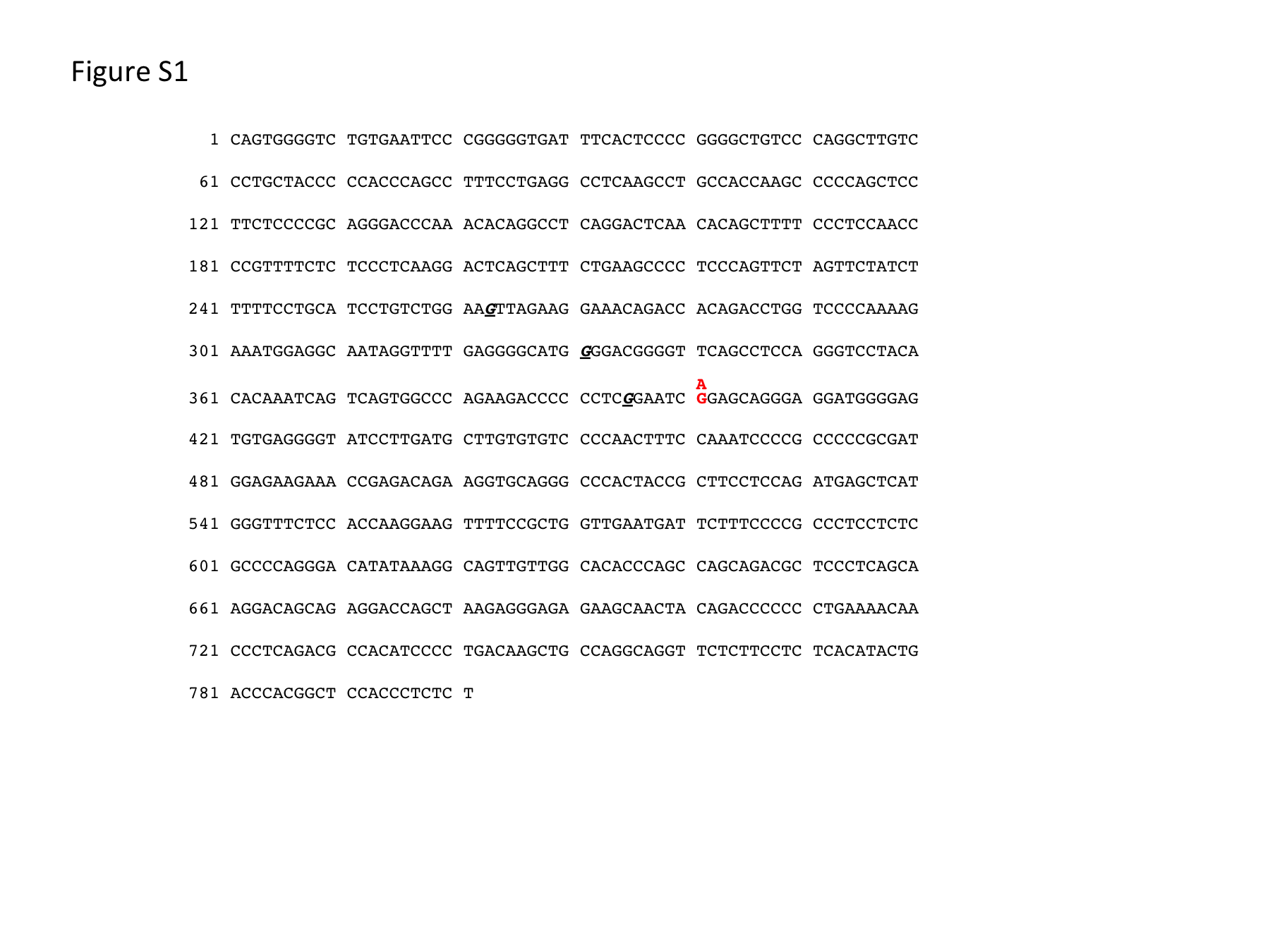

Supplement: Figure S1 — The nucleotide sequence of the promoter region of the TNF gene used in this study has been verified by sequencing. Polymorphic residues that have been previously been studied by Bayley at all as part of the same haplotype are highlighted (bold, italics, underlined). The -238 A/G variant that was the focus of this work is highlighted in red. (TIFF) [file pone.0076034.s001.tiff]
